# Binding of Soluble Ligands to Membrane Receptors: A Molecular Dynamics Simulation Study

**DOI:** 10.1021/acs.jpcb.5c01197

**Published:** 2025-07-12

**Authors:** Ruihan Hou, Jie Gao, Jingchun Chen, Rong Wang, Bartosz Różycki, Jinglei Hu

**Affiliations:** † Kuang Yaming Honors School, 12581 Nanjing University, Nanjing 210023, China; ‡ Department of Polymer Science and Engineering, Key Laboratory of High Performance Polymer Material and Technology of Ministry of Education, School of Chemistry and Chemical Engineering, Nanjing University, Nanjing 210023, China; § 86906 Institute of Physics, Polish Academy of Sciences, Aleja Lotników 32/46, Warsaw 02-668, Poland

## Abstract

The binding of membrane receptors to their ligands anchored in an apposing membrane mediates such biological processes as cell adhesion and signaling, and is known to be determined not only by direct receptor–ligand interactions but also by such factors as flexibility and thermal fluctuations of the apposing membranes. The binding of soluble ligands to membrane receptors initiates various cellular processes; however, to the best of our knowledge, its dependence on membrane properties has not been studied. Here, we employed molecular dynamics simulations to fill in this knowledge gap. Our simulations demonstrate that, in the absence of specific lipid–protein interactions and clustering of membrane receptors, the equilibrium and kinetic rate constants for the binding of soluble ligands to membrane receptors are determined almost entirely by direct receptor–ligand interactions and are practically unaffected by the curvature, flexibility and thermal fluctuations of the membrane.

## Graphical abstract

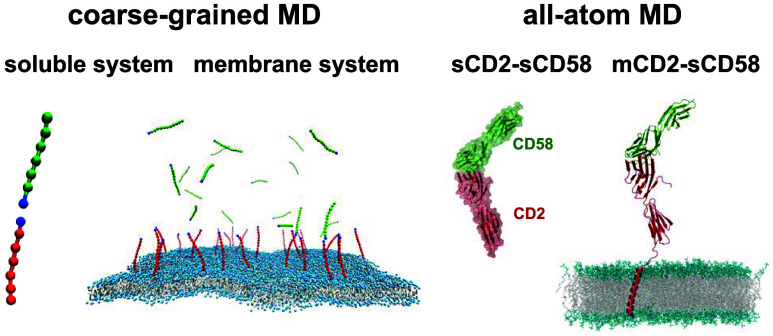

## Introduction

The binding of cell surface receptors to extracellular ligands enables cells to communicate with their surroundings and respond to specific stimuli, such as signal transduction,^1–3^ immune responses,^4,5^ and cancer metastasis.^6,7^ Quantifying the binding affinity between these proteins is therefore critically important for understanding their functional roles. Previous experimental^8–10^ and computational^11,12^ studies have characterized the binding affinity between membrane receptors (mR) and soluble ligands (sL) by measuring the binding equilibrium constants of soluble receptors (sR) and soluble ligands. However, these studies have largely neglected the influence of the membrane environment, which is a key factor present in cellular contexts. The assumption that mR-sL binding is equivalent to sR-sL binding remains untested, raising questions about the validity of extrapolating results from soluble systems to membrane-bound receptors.

The binding affinity between receptor and ligand proteins in solution is quantified by the standard binding equilibrium constant *K*_3D_ = [RL]_3D_/([R]_3D_[L]_3D_), where [RL]_3D_, [R]_3D_ and [L]_3D_ are the volumetric concentrations of the receptor–ligand complexes, free receptors, and free ligands in the three-dimensional (3D) solution, respectively. For membrane-bound receptors and ligands, an apparent two-dimensional (2D) binding equilibrium constant *K*_2D_ = [RL]_2D_/([R]_2D_[L]_2D_) has been introduced by analogy to *K*_3D_, assuming that the surface concentration of the membrane-bound receptor–ligand complexes [RL]_2D_ is proportional to the surface concentrations of the free receptors [R]_2D_ and ligands [L]_2D_ in the 2D membrane environment. Experimental measurements using different techniques, such as micropipette aspiration,^13,14^ flow chamber assays,^9,15^ and fluorescence spectroscopy,^10,16,17^ have yielded *K*_2D_ values that differ by several orders of magnitude for the same pairs of membrane-bound receptors and ligands, implying that *K*_2D_ may not be a constant. Theoretical modeling^18^ and molecular dynamics simulations^19–21^ have identified several factors that influence binding affinity in membrane environments. These include the average separation and thermal undulations of adhering membranes, as well as the association of adhesion proteins with lipid clusters within the membranes.^22–24^ These findings indicate that the linear proportionality assumed in the definition of *K*_2D_ is no longer valid. Consequently, the membrane environment needs to be carefully taken into account when using *K*_2D_ to quantify the binding affinity of membrane adhesion proteins. In systems where a membrane-bound receptor (mR) interacts with a soluble ligand (sL), the binding occurs at the interface between the 3D solution and the 2D fluid membrane. The central questions arising from this scenario are: Does the membrane environment influence the mR-sL binding? Is the mR-sL binding equivalent to sR-sL binding, or does it lie between the extremes of mR-mL and sR-sL binding? Addressing these questions is essential for a comprehensive understanding of receptor–ligand interactions in cellular contexts.

We developed a generic coarse-grained model for multidomain proteins and conducted GPU-accelerated molecular dynamics simulations to systematically study the interactions between mRs and sLs. This generic model allows for the flexible adjustment of key parameters, such as protein length, rigidity, and membrane curvature, enabling us to explore the binding kinetics in both membrane (mR-sL) and soluble systems (sR-sL). By analyzing approximately 5000 binding and unbinding events in each system, we find that the binding equilibrium and rate constants of mR-sL agree with those of sR-sL within less than 20%. This close agreement suggests that the binding mechanisms and kinetics of receptor–ligand interactions are highly similar between membrane and soluble systems, with no significant differences. To further validate these findings, we performed all-atom molecular dynamics simulations of single CD2-CD58 complexes in both membrane (mCD2-sCD58) and soluble (sCD2-sCD58) systems. The interaction between CD2, a T-cell surface receptor, and CD58, expressed on antigen-presenting cells, plays a critical role in the formation of the immunological synapse.^25–28^ Results from these simulations showed that the binding interface and binding free energy of CD2 and CD58 remain consistent between the soluble and membrane systems, supporting the findings from our coarse-grained molecular dynamics simulations. Our study provides, to the best of our knowledge, the first validation of characterizing mR-sL binding using sR-sL binding.

## Model and Methods

### Coarse-Grained Molecular Dynamics (CGMD) Simulations

We developed a generic coarse-grained model to study the binding of soluble ligands (sL) to membrane receptors (mR), by adapting the well-established Cooke-Deserno solvent-free lipid bilayer model^29^ (Figure 1a). Our coarse-grained model includes lipid, receptor (R), and ligand (L) molecules^30^ (Figure 1a). Each lipid consists of a hydrophilic head bead and two hydrophobic tail beads, linked by two finite extensible nonlinear elastic (FENE) bonds and straightened by a harmonic spring between the head bead and the second tail bead. Additionally, lipid tail beads experience attractive interactions which stabilize the bilayer structure. The R and L molecules are modeled as linear bead chains, where adjacent beads are connected via a harmonic potential, and consecutive three beads are governed by a bending potential. The strength *k*_bend_ of this bending potential regulates the conformational flexibility of Rs and Ls. The specific binding between Rs and Ls is described by a distance- and angle-dependent binding potential, ensuring a 1:1 binding stoichiometry. Any pairs of two beads experience a hard-core repulsive potential. MD simulations of the above coarse-grained model were performed using the Python GPU-Accelerated Molecular Dynamics software (PYGAMD).^31^ Further details of the CG model, force fields of different systems are provided in the Supporting Information (SI).

**Figure 1:**
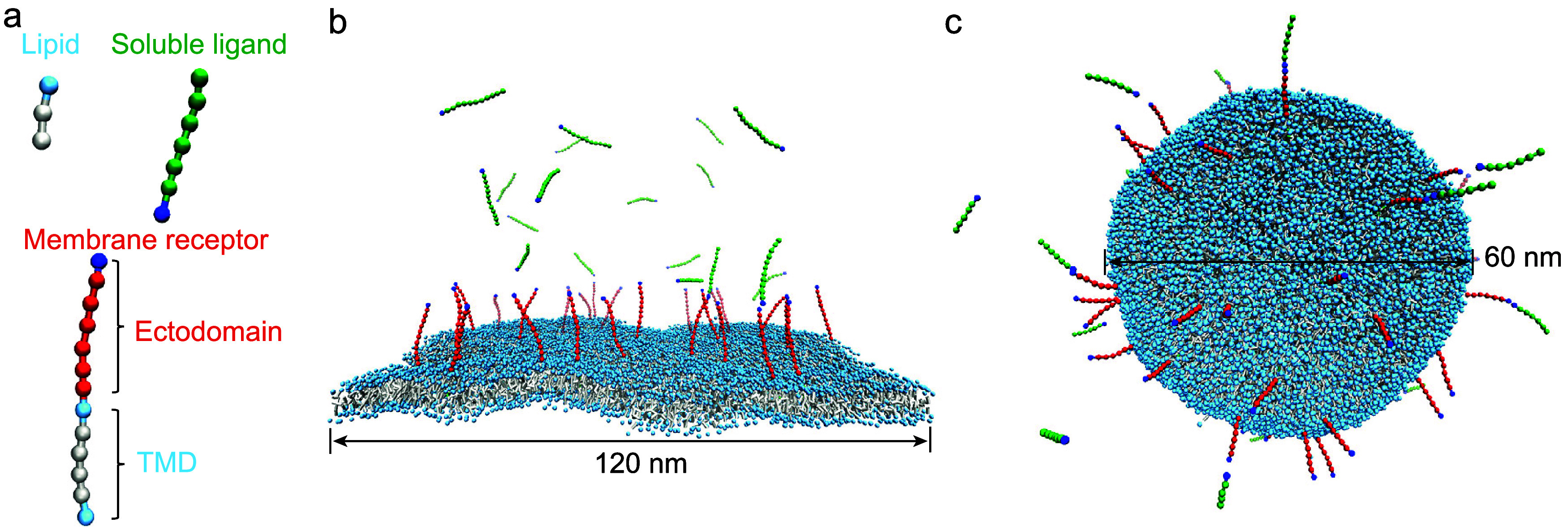
Coarse-grained model of membrane receptors binding to soluble ligands. (a) Molecular structure of the lipid, receptor, and ligand. The lipid is taken from the widely used implicit-solvent Cooke-Deserno model for fluid membranes,^29^ which includes one hydrophilic head bead (in light blue) and two hydrophobic tail beads (in gray). The receptor has an ectodomain (ECD) and a rigid transmembrane domain (TMD). The ligand shares the same structure as the receptor’s ECD. One receptor binds one ligand via the specific interaction between their binding sites (in blue). The flexibility of the receptor’s ECD and ligands can be easily tuned. (b) Quasi-planar and (c) vesicular membranes that exhibit thermal undulations and contain receptors binding to ligands. The snapshots in (b) and (c) are not drawn to scale.

### All-Atom Molecular Dynamics (AAMD) Simulations

We conducted AAMD simulations of a single CD2-CD58 complex in two systems (Figure 4a) using GROMACS 2023.3.^32^ The two systems were: (i) sCD2-sCD58 system where a soluble CD2 binds to a soluble CD58 in water without the lipid membrane or the transmembrane domain of CD2, and (ii) mCD2-sCD58 system where the membrane receptor CD2 forms the complex with soluble CD58. To explore the possible conformational changes of mCD2 upon binding, we also simulated a single membrane CD2 (mCD2) without CD58. Each system was subject to energy minimization using the steepest descent method, followed by 5 ns NVT equilibration at 300 K using the V-rescale thermostat^33^ and 5 ns NPT equilibration at 1 atm using the Berendsen barostat. Positional restraints with strength 1000 kJ mol^–1^ nm^–2^ ^34^ were applied to proteins during pre-equilibration. All hydrogen-containing bonds were constrained using the LINCS algorithm,^35^ and a 2 fs integration time step was used with the leapfrog integrator. Long-range electrostatics were computed via the PME method with a 1.0 nm cutoff,^36^ while van der Waals interactions were also truncated at 1.0 nm. Periodic boundary conditions were applied in all spatial directions. For statistical sampling, three independent NPT runs each of 500 ns were performed for all the simulated systems.

## Results and Discussion

We performed extensive molecular dynamics (MD) simulations of a generic coarse-grained (CG) model to investigate the binding of soluble ligands (sL) to membrane receptors (mR). Briefly, the fluid membrane is described by using the well-established implicit-solvent Cooke-Deserno model,^29^ where each lipid consists of a hydrophilic head bead and two hydrophobic tail beads (Figure 1a). The receptors (Rs) and ligands (Ls) are linear chains of beads. In addition to the ectodomain, each R contains a transmembrane domain. One R only binds specifically to one L via an angle- and distance-dependent potential (eq S6 in the SI) between their binding sites (blue beads in Figure 1a). Binding rate constants were determined from about 5000 binding and unbinding events observed in the CGMD simulations, a task that would be computationally infeasible using all-atom models.

The simulated systems differ in several key parameters: (i) the number, (ii) length, (iii) flexibility, and (iv) binding strength of Rs and Ls, as well as the (v) size and (vi) shape of the membrane. The contour length l of the R’s ectodomain and Ls was adjusted by varying the number of green and red beads per molecule (Figure 1a). Specifically, Rs and Ls with 6 and 11 green or red beads were considered, corresponding to l=6.65 and 11.4 σ_0_, respectively. Here, σ_0_ = 1 nm defines the basic length unit in the model. The flexibility of Rs and Ls was regulated by the strength *k*_bend_ of the bending potential governing bond angles (eq S5 in the SI). Two types of proteins were explored: (i) semirigid multidomain proteins, with *k*_bend_ = 10 ϵ_0_, and (ii) rigid rod-like proteins, with *k*_bend_ = 100 ϵ_0_, where ϵ_0_ is the basic energy unit of the CG model. The flexibility of the protein is described by an angular potential *V*_bend_(θ) = *k*_bend_[1 – cos­(θ – θ_0_)], where *k*_bend_ is the bending stiffness and θ is the bond angle with the preferred value of θ_0_. The strength of R-L binding potential was set to ϵ_bind_ = 14 and 15 ϵ_0_. We studied tensionless membranes of different shapes: (i) quasi-planar, (ii) supported planar, and (iii) vesicular. Quasi-planar and vesicular membranes (Figure 1b,c) have the bending rigidity of 13 *k*_B_ *T* according to ref 29. and undergo thermal undulations, while the supported planar membrane is nearly flat due to the suppression of undulations. Here, the absolute temperature *T* = 1.1 ϵ_0_/*k*_B_ with *k*_B_ the Boltzmann constant. All these systems with membrane receptors will be referred to as membrane systems. For comparison, we conducted MD simulations of soluble systems where the ligands bind to the ectodomain of the receptors in the absence of both the transmembrane domain and the lipid bilayer. Parameters of all simulated systems are detailed in the SI.

Figure 2 presents the primary results from our CGMD simulations. The binding rate constants *k*_on_ and *k*_off_ as well as the equilibrium constant *K* = *k*_on_/*k*_off_ were estimated through maximum likelihood analysis of the number of receptor–ligand complexes (RL) tracked over the course of simulations; see the SI for details. The constants *K*_3D_, *k*_on, 3D_, and *k*_off, 3D_ were measured from the soluble systems as previously described and used to rescale the constants obtained from the corresponding membrane systems. Remarkably, the rescaled equilibrium constant *K*/*K*_3D_ is close to unity over a wide range of RL concentrations, regardless of variations in the length l , flexibility *k*_bend_, and binding strength ϵ_bind_ of Rs and Ls, as well as the membrane’s shape and thermal undulations (Figure 2a). The rescaled on- and off-rate constants in Figure 2b were also found to approach unity. These results suggest that the receptor–ligand binding kinetics in the soluble and membrane systems are nearly equivalent, i.e., the membrane environment does not have a significant effect on the binding. In our CG model, the transmembrane domain of the receptors is rigid and does not undergo conformational changes upon binding, which is often the case for real membrane receptors.

**Figure 2:**
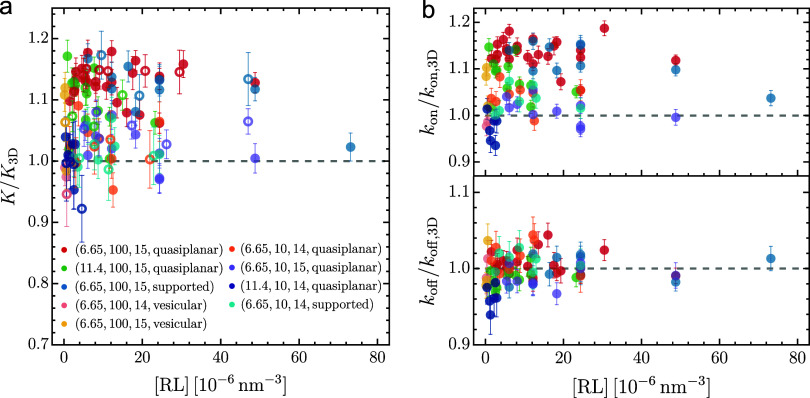
Rescaled binding equilibrium constant *K*/*K*_3D_ (a), and on- and off-rate constants *k*_on_/*k*_on, 3D_ and *k*_off_/*k*_off, 3D_ (b) versus volumetric concentration of receptor–ligand complexes [RL] as measured from CGMD simulations. The legend specifies the key parameters: contour length l , flexibility *k*_bend_, binding strength ϵ_bind_ of receptors and ligands, as well as membrane shape that vary across the simulated membrane systems. The constants *K*_3D_, *k*_on,3D_, and *k*_off,3D_ for ligand binding to the ectodomain of the receptor are obtained from simulations of the corresponding soluble systems.

For a single pair of receptor and ligand in a 3D solution, the binding kinetics and equilibrium are unchanged if the overall translation and/or rotation of the receptor is fixed while the ligand remains mobile. In the case of a membrane receptor interacting with a soluble ligand, the membrane restricts the translational mobility and overall rotation of the receptor. However, if the membrane does not obstruct the binding interface or induce conformational changes in the receptor and ligand, and the receptors have no propensity to cluster, the binding of the membrane receptor to the soluble ligand (mR-sL) should be equivalent to that of the soluble receptor and soluble ligand (sR-sL). It is worth noting that receptor clustering can promote local rebinding events: a ligand that dissociates from one receptor may rebind to a neighboring receptor, effectively increasing the apparent on-rate constant *k*_on_. This rebinding effect arising from clustering, which is absent in bulk solution, is not captured in our current model.

We now examine the binding interface and possible conformational changes of membrane receptors upon ligand binding. Figure 3a,b show the distributions of the binding angles θ_1_ and θ_2_, as well as the distribution of the distance *r* between the binding sites within receptor–ligand complexes. The distributions observed in the membrane system are identical to those in the soluble system, indicating that the binding interface is governed by the binding potential and is unaffected by the presence of the membrane. Figure 3c illustrates the distribution of the end-to-end distance *R*_e_ of the receptor’s ectodomain before and after binding to ligands. In the soluble system, the distributions before and after binding are identical, as the binding of soluble receptors and ligands does not induce conformational changes in our coarse-grained model. In contrast, the distribution of *R*_e_ after binding is narrower than before in the membrane system, suggesting that the semirigid receptors experience a loss of conformational entropy upon binding in the membrane environment. This observation aligns with the result shown in Figure 2a, where semirigid receptors (*k*_bend_ = 10 ϵ_0_) seem to exhibit slightly smaller binding constants *K* with ligands compared to rigid receptors (*k*_bend_ = 100 ϵ_0_).

**Figure 3:**
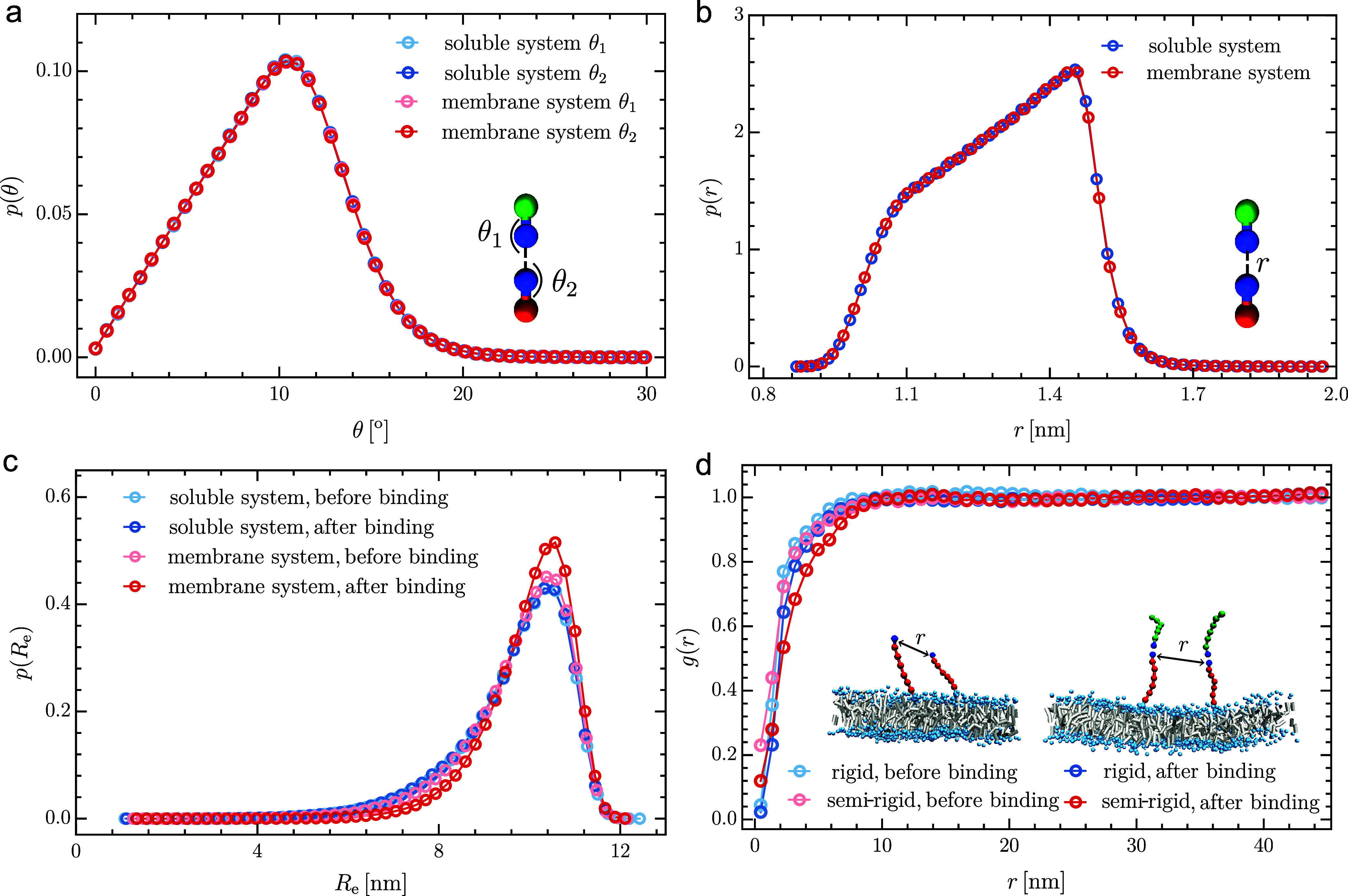
Analysis of binding interface, receptor conformation and lateral receptor-receptor interactions from CGMD simulations of quasi-planar membrane systems and the corresponding soluble systems. (a) Distribution of binding angle and (b) distribution of binding distance for the receptor–ligand complexes. (c) Distribution of the end-to-end distance *R*_e_ of the receptor’s ectodomain before and after binding. (d) Pair correlation function between the binding sites of two membrane receptors before and after binding. Simulation parameters: l = 11.4 nm, *k*_bend_ = 10 ϵ_0_, ϵ_bind_ = 14 ϵ_0_, *A*_me_ = 90 × 90 nm^2^ for (a–c); and *N*_R_ = *N*_L_ = 9, l = 6.65 nm, ϵ_bind_ = 40 ϵ_0_, *A*_me_ = 90 × 90 nm^2^ for (d).

We further quantified the lateral interactions between membrane receptors before and after ligand binding by analyzing their pair correlation functions. Figure 3d shows the pair correlation function *g*(*r*) between the binding sites of two membrane receptors. For both semirigid and rigid receptors, *g*(*r*) < 1 at distances smaller than approximately 10 nm, which is comparable to the contour length of the receptors. This indicates the presence of short-range repulsive interactions between the receptors. Semirigid receptors exhibit slightly stronger repulsion after ligand binding, which restricts their conformational flexibility, as evidenced by the narrower distribution of the end-to-end distance *R*_e_ in Figure 3c. At larger distances, *g*(*r*) ≈ 1, suggesting that long-range membrane-mediated interactions are negligible. Overall, the lateral interactions between membrane receptors exhibit no significant difference before and after ligand binding.

To complement our study based on the coarse-grained model and to gain atomistic insights into the binding interactions, we performed AAMD simulations of a single CD2-CD58 complex in both soluble (sCD2-sCD58) and membrane (mCD2-sCD58) systems (Figure 4a). These simulations allowed us to characterize the influence of the membrane environment on the binding interactions. Figure 4b shows the time series of the contact surface areas (CSA) between CD2 and CD58 for both systems. This CSA, which accounts for the reduction in the solvent accessible surface area of the two proteins upon binding, fluctuates around its average value over the 500 ns simulation period. The time-averaged CSA values are 6.8 ± 1.0 nm^2^ for the soluble system and 7.2 ± 0.7 nm^2^ for the membrane system. Figure 4c indicates that the average number of hydrogen bonds formed between CD2 and CD58 is approximately 12 in both systems, with the average hydrogen bond lifetimes remaining consistent within statistical error, at around 350 ps. Energy decomposition analysis further reveals that the key residues at the CD2-CD58 binding interface are identical in both systems (Figure S2). Together, these findings suggest that the presence of the membrane has a negligible effect on the binding interface.

**Figure 4:**
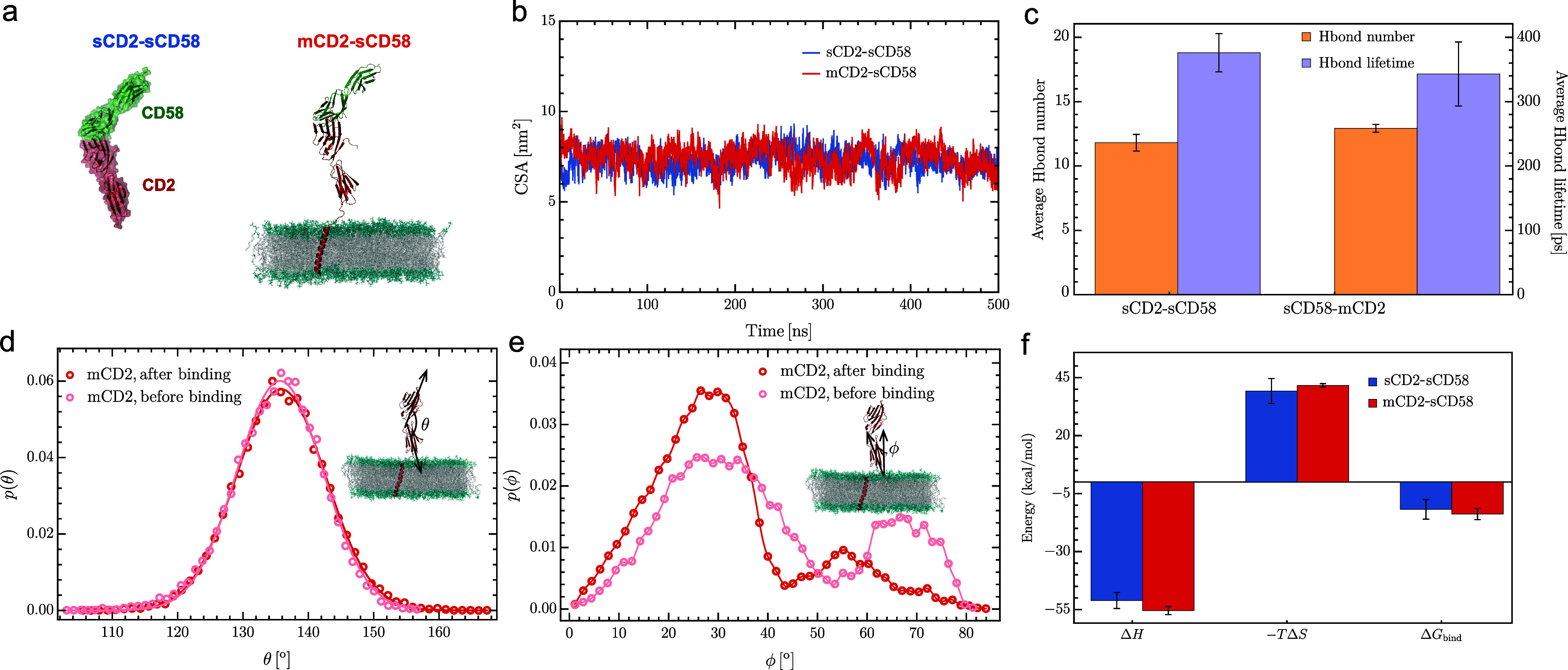
All-atom MD simulations of the CD2-CD58 complex. (a) Snapshots of the soluble and membrane systems. In the soluble system, a soluble CD2 lacking the transmembrane domain binds a soluble CD58. In the membrane system, a membrane-bound CD2 forms a complex with a soluble CD58. The systems contain ∼ 140,000 and ∼ 420,000 atoms, respectively. Water is not displayed for clarity. (b) Contact surface area (CSA) between CD2 and CD58 over time. (c) Average number and lifetime of hydrogen bonds formed between CD2 and CD58 in the complex. (d) Distribution of the angle θ between CD2’s two extracellular domains (ECDs). (e) Distribution of the tilt angle ϕ of CD2’s membrane-proximal ECD relative to the local membrane normal. (f) Decomposition of the binding free energy in both systems. The error bars in (c) and (f) are standard deviations computed from three independent runs each of 500 ns (Figure S2).

To further investigate the conformational changes of mCD2 upon binding to sCD58, we analyzed the distributions of two angles, θ and ϕ, as illustrated in Figure 4d,e. The angle θ is defined by the long principal axis of CD2’s two extracellular domains (ECDs) and reflects the flexibility of the interdomain linker. The angle ϕ represents the tilt of CD2’s membrane-proximal ECD relative to the local membrane normal. Figure 4d shows that the distribution of the orientation angle θ for mCD2 remains largely unchanged before and after binding to sCD58. Figure 4e demonstrates that free mCD2 exhibits a slightly wider distribution of the tilt angle ϕ, indicating that it adopts a broader range of orientations relative to the membrane than mCD2 bound to sCD58. We also estimated the binding free energies for both systems using the MM/GBSA method^37–40^ (see the SI for details). As shown in Figure 4f, the binding free energies for the sCD2-sCD58 and mCD2-sCD58 systems are Δ*G*_bind_ = −11.8 ± 4.2 and −13.8 ± 2.4 kcal·mol^–1^, respectively. For reference, the experimental measurement of sCD2-sCD58 binding reported a value of −7.1 kcal mol^–1^.^41^ Given the inherent approximations of the MM/GBSA method, the agreement with the experimental result is reasonably close. To further investigate the role of membrane composition in receptor–ligand interactions, we performed AAMD simulations of the mCD2-sCD58 system in a mixed bilayer composed of POPC and cholesterol at a 7:3 molar ratio. For a single receptor–ligand pair, we observed no significant differences in binding behavior compared to the pure POPC membrane (Figure S4). In summary, the AAMD simulations of single CD2-CD58 complexes suggest that the membrane environment does not significantly alter the binding interactions between receptors and soluble ligands. Although the present study focuses on the mCD2-sCD58 system, it is important to recognize that under physiological conditions, both CD2 and CD58 are anchored on opposing cell surfaces. In such membrane-anchored systems, receptor–ligand binding is characterized by *K*_2D_, rather than *K*_3D_. These two constants differ not only in units but also in their physical interpretations. Previous studies^19–24,42–47^ have shown that thermal undulations and heterogeneity of the membranes significantly influence both *K*_2D_ and adhesion dynamics. We would also like to note that capturing the effects of membrane crowding and receptor clustering requires simulations of the mCD2-mCD58 system on large time and length scales that are currently beyond the reach of AAMD. Moreover, explicit-water simulations of this system would be strongly affected by the choice of the number of water molecules between the membranes. Recently, we have developed a multiscale approach that combines CGMD simulations with a dynamic Monte Carlo framework to efficiently explore the large time and length scales relevant to membrane adhesion.^30^ This approach is well-suited for studying the mCD2-mCD58 system and can be applied in our future work.

## Conclusions

In this study, we investigated the binding interactions between membrane-bound receptors (mRs) and soluble ligands (sLs) using a combination of coarse-grained and all-atom molecular dynamics simulations. Our results show that the binding affinity and kinetics of mR-sL interactions are highly similar to those of sR-sL interactions, with no significant differences in the equilibrium constants or rate constants between the two systems (Figure 2). The binding interface and energy profiles observed in both the membrane and soluble systems were found to be consistent, supporting the conclusion that the membrane environment has minimal impact on the binding of receptors to soluble ligands.

Furthermore, we observed that membrane receptors experience subtle conformational changes upon ligand binding, with semirigid receptors displaying a loss of conformational entropy in the membrane environment (Figure 3). However, the lateral interactions between membrane receptors were not significantly altered by ligand binding, and the binding interface remained unchanged (Figure 4). These results highlight the robustness of receptor–ligand binding interactions, even in the context of complex membrane environments, and provide valuable insights into the role of membranes in modulating binding kinetics. In addition, the current model does not account for lipid heterogeneity, receptor clustering, or signal-associated conformational transitions, all of which may play critical roles in the signal transduction of many biological receptors such as G protein-coupled receptors and receptor tyrosine kinases by influencing receptor spatial organization and binding dynamics.^48–55^ Overall, this study offers a comprehensive validation of using soluble receptor–ligand binding to describe membrane-bound receptor–ligand interactions, enhancing our understanding of cellular adhesion and signaling processes.

## Supplementary Material



## Data Availability

The input files used in the CGMD and AAMD simulations are available at https://github.com/Houruihan/soluble_ligand_binding.

## Notes

Three sections provide details on CGMD and AAMD simulations, as well as the estimation of binding rate constants using maximum likelihood analysis; the parameters of the simulated membrane systems (Tables S1–S3); binding constants for soluble ligands binding to soluble receptors, obtained from CGMD simulations (Table S4); dwell-time probability distribution obtained from CGMD simulations (Figure S1); illustration of the results of AAMD simulations of the CD2-CD58 complex in both membrane and soluble systems (Figure S2); energy decomposition per residue for CD2-CD58 interactions in soluble and membrane systems based on AAMD simulations (Figure S3); results of the CD2-CD58 complex bound to a membrane composed of POPC and cholesterol (Figure S4) (PDF)
