# Supplementary material for: Binding of Soluble Ligands to Membrane Receptors: A Molecular Dynamics Simulation Study
Source: J Phys Chem B. 2025 Jul 12;129(29):7475–82. doi: 10.1021/acs.jpcb.5c01197 (PMC12302211; doi:10.1021/acs.jpcb.5c01197)
Supplement: Supplementary file 1 [file jp5c01197_si_001.pdf]

Supporting Information for "Binding of Soluble Ligands to Membrane Receptors: A Molecular Dynamics Simulation Study"

Ruihan Hou,[†,‡,§] Jie Gao,[†,‡,§] Jingchun Chen,[†] Rong Wang,[‡] Bartosz Różycki,[*,¶] and Jinglei Hu[*,†,‡]

†*Kuang Yaming Honors School, Nanjing University, Nanjing 210023, China*

‡*Department of Polymer Science and Engineering, Key Laboratory of High Performance Polymer Material and Technology of Ministry of Education, School of Chemistry and Chemical Engineering, Nanjing University, Nanjing 210023, China*

¶*Institute of Physics, Polish Academy of Sciences, Aleja Lotników 32/46, 02-668 Warsaw, Poland*

§*Contributed equally to this work*

E-mail: rozycki@ifpan.edu.pl; hujinglei@nju.edu.cn

Coarse-grained molecular dynamics (CGMD) simulations

Model

In the coarse-grained model, the lipid is adopted from the widely-used implicit-solvent model for fluid membranes by Cooke and Deserno,[1] where each lipid contains one hydrophilic head bead (LH) and two hydrophobic tail beads (LT). A receptor (R) consists of a transmembrane domain (TMD) and extracellular domains (ECDs) which include extracellular beads (PE,

shown in red) and a binding site (PB, shown in blue) (Fig. 1a). The TMD of each R contains four hydrophobic lipid-tail-like beads (PT, in dark gray) and two hydrophilic lipid-head-like beads (PH, in blue). Each ligand (L) has the same molecular structure as that of the R's ECDs. Any pairs of two beads experience the hard-core repulsion modeled by the purely repulsive potential

$$V_{\rm rep}(r) = \begin{cases} 4\,\epsilon_{\rm rep}\left[\left(\frac{\sigma}{r}\right)^{12} - \left(\frac{\sigma}{r}\right)^{6} + \frac{1}{4}\right], & r \le 2^{1/6}\,\sigma \\ 0, & r > 2^{1/6}\,\sigma \end{cases} \tag{S1}$$

where $\sigma = \sigma_0$ and $\epsilon_{\rm rep} = \epsilon_0$ for most of the pairs. Here, σ_0 is the basic length unit and ϵ_0 the basic energy unit. For LH-LH and LH-LT pairs, $\sigma = 0.95\,\sigma_0$. For PT-LH and PH-LT pairs, $\epsilon_{\rm rep} = 5\,\epsilon_0$. To ensure the R-L binding with 1:1 stoichiometry, $\sigma = 1.5\,\sigma_0$ and $\epsilon_{\rm rep} = 10\,\epsilon_0$ are chosen for PB-PB pairs that belong to two R or L molecules.

Adjacent beads within the R or L molecules are connected via the harmonic potential

$$V_{\rm bond}(r) = \frac{1}{2}\,k_{\rm bond}\,(r - r_0)^2 \tag{S2}$$

with the spring constant $k_{\rm bond} = 100\,\epsilon_0/\sigma_0^2$ and the rest length $r_0 = 0.95\,\sigma_0$. In each lipid, the LH and the last LT beads are also bonded via this potential with $k_{\rm bond} = 10\,\epsilon_0/\sigma_0^2$ and $r_0 = 4\,\sigma_0$. Additionally, any two consecutive beads of each lipid are linked by the finite extensible nonlinear elastic (FENE) bond

$$V_{\rm FENE}(r) = -\frac{1}{2}\,k_{\rm FENE}\,r_\infty^2 \log[1 - (r/r_\infty)^2] \tag{S3}$$

with the stiffness $k_{\rm FENE} = 30\,\epsilon_0/\sigma_0^2$ and the divergence length $r_\infty = 1.5\,\sigma_0$.

The lipid tail beads experience pairwise attractive potentials

$$V_{\text{att}}(r) = \begin{cases} -\epsilon_{\text{att}}, & r < r_{\text{c}} \\ -\epsilon_{\text{att}} \cos^2[\pi(r - r_{\text{c}})/(2w_{\text{c}})], & r_{\text{c}} \leq r \leq r_{\text{c}} + w_{\text{c}} \\ 0, & r > r_{\text{c}} + w_{\text{c}} \end{cases} \tag{S4}$$

where $r_{\text{c}} = 2^{1/6}\,\sigma_0$, $w_{\text{c}} = 1.6\,\sigma_0$, and $\epsilon_{\text{att}} = \epsilon_0$ are chosen to obtain a stable fluid bilayer with the bending rigidity of about $13\,k_{\text{B}}T$ at room temperature,[1] which is within the experimental range of about 10 to 40 $k_{\text{B}}T$.[2,3] k_{B} is the Boltzmann constant and T the absolute temperature. This attraction effectively accounts for the hydrophobic interactions of lipid molecules. Any pairs of an R's or L's lipid-tail-like bead and a lipid tail bead, i.e., PT-LT pairs, also interact through this potential to facilitate the insertion of the R's TMD into the bilayer.

To capture the conformational flexibility of R and L molecules, every three adjacent beads in each R and L interact via the bending potential

$$V_{\text{bend}}(\theta) = k_{\text{bend}}[1 - \cos(\theta - \theta_0)], \tag{S5}$$

where k_{bend} is the strength and θ_0 is the preferred angle. For R's ECD beads and L's beads (red, green, and blue beads in Fig. 1a), $\theta_0 = 180°$. $k_{\text{bend}} = 100\,\epsilon_0$ is chosen to obtain a rigid rodlike protein, while $k_{\text{bend}} = 10\,\epsilon_0$ for a semi-rigid protein. For TMD beads and PE-PH-PT of each R, $k_{\text{bend}} = 100\,\epsilon_0$ and $\theta_0 = 180°$ are set to maintain a rigid linear structure of TMDs within the lipid bilayers.

The specific binding of R and L is modeled via the distance- and angle-dependent binding potential

$$U_{\text{bind}}(r, \theta_1, \theta_2) = V_{\text{bind}}(r) f_1(\theta_1) f_2(\theta_2), \tag{S6}$$

where the radial part $V_{\rm bind}(r)$ assumes

$$V_{\rm bind}(r) = \begin{cases} -\epsilon_{\rm bind} - u_{\rm c}, & r \leq 2^{1/6}\sigma + w_{\rm f} \\ 4\,\epsilon_{\rm bind}\left[\left(\frac{\sigma}{r-w_{\rm f}}\right)^{12} - \left(\frac{\sigma}{r-w_{\rm f}}\right)^{6}\right] - u_{\rm c}, & 2^{1/6}\sigma + w_{\rm f} < r < 2.5\sigma + w_{\rm f} \\ 0, & r \geq 2.5\sigma + w_{\rm f} \end{cases} \tag{S7}$$

with $\sigma = 0.95\,\sigma_0$, $w_{\rm f} = 0.4\,\sigma$, $\epsilon_{\rm bind} = 15\,\epsilon_0$, and $u_{\rm c} = 4\epsilon_0[(1/2.5)^{12} - (1/2.5)^6] \approx -0.016\,\epsilon_0$. The angular part $f_i(\theta_i)$ with $i = 1, 2$ takes the form

$$f_i(\theta_i) = \begin{cases} 1, & \theta_i \leq \theta_{i,0} \\ e^{-\frac{1}{2}k_i(\theta_i - \theta_{i,0})^2}, & \theta_i > \theta_{i,0} \end{cases} \tag{S8}$$

with $\theta_{1,0} = \theta_{2,0} = 10^\circ$ and $k_1 = k_2 = 15\,\text{rad}^{-2}$. The angles θ_1 and θ_2 are defined by the two binding sites (blue) of a pair of receptor and ligand molecules and the neighboring beads in the receptor (red) and ligand (green), as shown in Fig. 3a. Specifically, θ_1 is defined for the ligand by the two blue beads and the green bead. $\theta_1 = 0^\circ$ corresponds to a colinear arrangement of the three beads. θ_2 is defined analogously for the receptor.

Simulations

MD simulations of our coarse-grained model were performed using the Python GPU-Accelerated Molecular Dynamics software (PYGAMD).[4] MD runs in the canonical ensemble were conducted within a rectangular box of volume $V_0 = L_x \times L_y \times L_z$ under periodic boundary conditions. A constant temperature $T = 1.1\,\epsilon_0/k_{\rm B}$ was maintained using a Langevin thermostat with a drag coefficient $\gamma = \epsilon_0 t_0/\sigma_0^2$, where t_0 is the basic time unit. The lipid membrane was initially assembled and oriented on average parallel to the xy-plane, and thus had a project area of $A_{\rm me} = L_x \times L_y$.

As described in the main text, the simulated systems differ in the number ($N_{\rm R}$ and $N_{\rm L}$), length ℓ, molecular flexibility $k_{\rm bend}$, and binding strength $\epsilon_{\rm bind}$ of Rs and Ls, as well as the

size (projected area A_{me}) and shape (quasi-planar, supported planar, and vesicular) of the membrane. All the simulated membrane systems are listed in Tabs. S1-S3.

The extension of the box in the direction perpendicular to the membranes $L_z = 100$ nm was chosen for all systems. The number of lipids in each membrane was adjusted such that the membrane tension vanishes, corresponding to the area per lipid of approximately $1.2\,\sigma_0^2$. Matching the thickness of the simulated bilayer about $5\,\sigma_0$ to the experimental value of 5 nm leads to the physical length scale $\sigma_0 \approx 1$ nm. The physical time scale $t_0 \approx 10$ ns was obtained by mapping the lateral diffusion coefficient of the lipids $0.012\,\sigma_0/t_0^2$ to the typical value of 1 µm^2/s. The integration time step was set to $\delta t = 0.01\,t_0 \approx 0.1$ ns. For each system, a relaxation run of $2 \times 10^7\,\delta t$ was performed for thermal equilibration and a subsequent run of up to $4 \times 10^9\,\delta t$ was conducted for statistical sampling. From these simulations, approximately 5000 binding and unbinding events were observed, and binding rates and equilibrium constants were extracted using the maximum likelihood method as will be described below.

To measure the binding equilibrium and rate constants of Rs and Ls in the absence of the membrane environment, four systems with sR and sL that both lack the TMDs were simulated. In these systems, 10 pairs of sR and sL were enclosed in a cubic box of side length $L = 100$ nm. Each system was subjected to a relaxation run of $5 \times 10^7\,\delta t$ and a production run of up to $5 \times 10^9\,\delta t$ for data acquisition, during which over 5000 binding and unbinding events were recorded to determine the binding constants. Table S4 shows the binding equilibrium and rate constants measured from these systems.

All-atom molecular dynamics (AAMD) simulations

AAMD simulations of a CD2-CD58 complex were conducted by using the GROMACS 2023.3 package.[5] The amber ff14SB[6–8] force field was applied for CD2 and CD58. The structure of extracellular domains for CD2 and CD58 were obtained from the PDB ID 1HNF[9] and

PDB ID 1CCZ,[10] respectively. The binding interface was modeled according to the PDB ID 1QA9,[11] which contains the binding domain of CD2 and CD58. The CD2-CD58 complex was solvated with TIP3P water, keeping the distance between the box and proteins at 1.5 nm. Counter ions Na^+ and Cl^{-1} were added to neutralize the system and the concentration was set at 0.15 M. For the membrane-bound CD2, its TMD was built as standard Alpha-helices, while the linker regions between the TMD and ECDs were modeled by the online server ModLoop.[12] The CD2-CD58 complex was embedded in either a pure POPC bilayer or a POPC:cholesterol (7:3) bilayer, generated via CHARMM-GUI .[13] POPC and cholesterol molecules were parameterized using the AMBER Lipid21 force field.

The soluble system (sCD2-sCD58) contains 142,580 atoms enclosed within a rectangular box of dimension $10 \times 10 \times 15$ nm^3, whereas the membrane system (mCD2-sCD58) includes 421,508 atoms within a $13 \times 13 \times 23$ nm^3 box. As a control, the system with a single membrane-bound CD2 (mCD2) consists of 346,758 atoms within a $13 \times 13 \times 19$ nm^3 box. The cholesterol-containing membrane system (mCD2-sCD58-chol) comprises 376,741 atoms in a $12\times12\times27$ nm^3 box, with a membrane composed of POPC and cholesterol at a 7:3 ratio. For statistical sampling, three independent NPT runs each of 500 ns were performed for all the simulated systems, except for the cholesterol-containing system, which was simulated for 200 ns in each run.

Computational approaches are commonly employed to estimate the binding free energy of protein-protein interactions. Methods such as free energy perturbation[14,15] and umbrella sampling[16–18] can compute free energy differences with high accuracy. However, these approaches often face convergence issues[19,20] and are computationally demanding.[21] As a compromise between accuracy and computational cost, the Molecular Mechanics/Generalized Born Surface Area (MM/GBSA) method has been widely adopted in studies of protein-ligand and protein-protein interactions.[22–24] We utilized the MM/GBSA method to calculate the binding free energy and employed normal mode analysis to estimate the entropic contribution. In MM/GBSA, the binding free energy ΔG_{bind} between the receptor and ligand can

be decomposed as follows:[23–26]

$$\begin{aligned} \Delta G_{\text{bind}} &= \Delta H - T\Delta S = \Delta E_{\text{MM}} + \Delta G_{\text{sol}} - T\Delta S \\ \Delta E_{\text{MM}} &= \Delta E_{\text{int}} + \Delta E_{\text{ele}} + \Delta E_{\text{vdw}} \\ \Delta G_{\text{sol}} &= \Delta G_{\text{GB}} + \Delta G_{\text{SASA}} \end{aligned} \tag{S9}$$

where ΔE_{MM} represents the free energy difference in the gas phase, which can be decomposed as internal energy ΔE_{int}, electrostatic interaction energy ΔE_{ele} and van der Waals interaction energy ΔE_{vdw}. ΔG_{sol} corresponds to the solvation free energy, including polar part ΔG_{GB} and nonpolar part ΔG_{SASA}. The electrostatic term ΔE_{ele} is generally calculated according to

$$\Delta E_{\text{ele}} = \sum_i \sum_j \frac{q_i q_j}{4\pi\varepsilon_0\varepsilon_{\text{in}} r_{ij}} \tag{S10}$$

where q_i was the charge of atom i in one protein, while q_j is the charge of atom j in the other protein. r_{ij} is the distance between the atoms i and j, ε_0 and ε_{in} were the dielectric constant in vacuum and the relative dielectric constant of the solute respectively. Notably, when the proteins carry net charges, there will be many counter ions around them, which gives rise to the great change of the effective electrostatic interaction between them. The calculation of ΔE_{ele} has been shown to be improved by using a larger dielectric constant, and the choice of $\epsilon_{\text{in}} = 2$–4 has been recommended.[27–29] $\epsilon_{\text{in}} = 2$ was chosen in our calculations. The entropic contribution $-T\,\Delta S$ was estimated using the normal mode analysis.[30] Specifically, to calculate the binding free energy, 500 frames were sampled from the last 100 ns with an interval of every 200 ps to calculate the binding free energy via MM/GBSA.

Estimation of binding rate constants from maximum likelihood analysis

We briefly review the maximum likelihood analysis for extracting the binding kinetics from molecular dynamics simulation trajectories.[31,32] The R-L binding and unbinding events divide the trajectories into different states with different numbers of R-L complexes. The system with N_{R} receptors and N_{L} ligands has totally $(N+1)$ states with $N = \min(N_{\mathrm{R}},\, N_{\mathrm{L}})$ the maximum number of R-L complexes. The simulation trajectories can be mapped to a Markov model

$$0 \underset{k_-^{(1)}}{\overset{k_+^{(0)}}{\rightleftharpoons}} 1 \underset{k_-^{(2)}}{\overset{k_+^{(1)}}{\rightleftharpoons}} 2 \underset{k_-^{(3)}}{\overset{k_+^{(2)}}{\rightleftharpoons}} 3 \cdots N-1 \underset{k_-^{(N)}}{\overset{k_+^{(N-1)}}{\rightleftharpoons}} N, \tag{S11}$$

where the transition rates $k_+^{(n)}$ and $k_-^{(n)}$ are, repectively, related to the on- and off-rate constants $k_{\mathrm{on}}^{(n)}$ and $k_{\mathrm{off}}^{(n)}$ via

$$k_+^{(n)} = (1/A)\,(N_{\mathrm{L}} - n)\,(N_{\mathrm{R}} - n)\,k_{\mathrm{on}}^{(n)}, \tag{S12}$$

and

$$k_-^{(n)} = n k_{\mathrm{off}}^{(n)}. \tag{S13}$$

The equilibrium constant that characterizes the R-L binding affinity is defined as

$$K_{\mathrm{2D}}^{(n)} = \frac{k_{\mathrm{on}}^{(n-1)}}{k_{\mathrm{off}}^{(n)}}. \tag{S14}$$

The on- and off-rate constants $k_{\mathrm{on}}^{(n)}$ and $k_{\mathrm{off}}^{(n)}$ in Eqs. S12-S13 can be determined from the observed numbers of transitions between the states and from the overall dwell times in the states. The binding and unbinding events divide the simulation trajectories into time

windows i of length t_i in state n_i, which are followed by a transition into state $n_i + s_i$ with $s_i = 1$ or -1. The probability for staying in state n_i for a dwell time t_i is $P_{n_i}(t_i) = \exp\{-[k_+^{(n_i)} + k_-^{(n_i)}]t_i\}$. We have analyzed all the trajectories, and Fig. S1 presents an example from the system with 18 membrane receptors and 18 soluble ligands anchored to a 60× 60 nm^2 membrane. Fig. S1a shows the number of receptor-ligand bonds n as a function of time t for a short time interval. Fig. S1b shows that the dwell times for the most probable state ($n = 3$) follow an exponential distribution, consistent with memoryless (Markovian) behavior. The probability for the time window i with the observed transition is $p_i \propto P_{n_i}(t_i) \cdot k_+^{(n_i)}$ for $s_i = 1$ and $p_i \propto P_{n_i}(t_i) \cdot k_-^{(n_i)}$ for $s_i = -1$. The likelihood function is the probability of the whole trajectory and takes the form

$$L = \prod_i p_i = \prod_{n=0}^{N} \left[k_+^{(n)}\right]^{N_n^+} \left[k_-^{(n)}\right]^{N_n^-} e^{-\left[k_+^{(n)}+k_-^{(n)}\right]T_n}, \tag{S15}$$

where N_n^+ is the total number of transitions from state n to $n+1$, N_n^- the total number of transitions from state n to $n-1$, and T_n the total dwell time in state n.

Maximizing the likelihood function L in Eq. S15 with respect to the rate constants $k_{\rm on}^{(n)}$ and $k_{\rm off}^{(n)}$ leads to the maximum likelihood estimators

$$k_{\rm on}^{(n)} = \frac{N_n^+ V}{(N_{\rm R} - n)(N_{\rm L} - n)T_n} \tag{S16}$$

and

$$k_{\rm off}^{(n)} = \frac{N_n^-}{nT_n}. \tag{S17}$$

Our estimator for the binding constant defined in Eq. S14 is

$$K_{\rm 2D}^{(n)} = \frac{nVT_n}{(N_{\rm R} - n + 1)(N_{\rm L} - n + 1)T_{n-1}}. \tag{S18}$$

For each simulation, we record the numbers of transitions N_n^+ and N_n^- as well as the overall dwell times in each state T_n, and estimate the constants in each state according to Eqs. S16-S18. For membrane systems, the effective volume accessible to the receptors and ligands, V, is determined by subtracting the membrane-occupied volume from the total system volume $V_0 = L_x \times L_y \times L_z$. Since the physical processes such as receptor diffusion, membrane thermal fluctuations, and stochastic rebinding are already included in the simulation trajectories, our maximum likelihood analysis of binding kinetics will automatically reflect the effects of these processes.

Table S1: Parameters of the 28 quasi-planar membrane systems considered in CGMD simulations.

ℓ (nm)	$\epsilon_{\rm bind}$ (ϵ_0)	$k_{\rm bend}$ (ϵ_0)	$N_{\rm R}$	$N_{\rm L}$	L_x, L_y (nm)
6.65	15	100	8	4	30
			8	8	30
			18	9	60
			18	18	60
			25	15	90
			25	25	90
			32	16	120
			32	32	120
	15	10	8	4	30
			8	8	30
			18	9	60
			18	18	60
			25	15	90
			25	25	90
	14	10	8	4	30
			8	8	30
			18	18	60
			25	25	90
	40	10	9	9	90
		100	9	9	90
11.4	15	100	8	4	30
			8	8	30
			18	18	60
			25	25	90
	14	10	8	4	30
			8	8	30
			18	18	60
			25	25	90

Table S2: Parameters of the 8 supported-planar membrane systems considered in CGMD simulations.

ℓ (nm)	ϵ_{bind} (ϵ_0)	k_{bend} (ϵ_0)	N_{R}	N_{L}	L_x, L_y (nm)
6.65	15	100	8	4	30
			8	8	30
			18	18	60
			25	25	90
	14	10	8	4	30
			8	8	30
			18	18	60
			25	25	90

Table S3: Parameters of the two vesicular membrane systems considered in CGMD simulations. d is the outer diameters of the vesicular membrane.

ℓ (nm)	ϵ_{bind} (ϵ_0)	k_{bend} (ϵ_0)	N_{R}	N_{L}	d (nm)
6.65	15	100	40	40	60
	14	100	40	40	50

Table S4: Binding equilibrium and rate constants of soluble ligands binding to soluble receptors in 3D solution measured from CGMD simulations.

ℓ (nm)	ϵ_{bind} (ϵ_0)	k_{bend} (ϵ_0)	K_{3D} (nm^3)	$k_{\mathrm{on,\ 3D}}$ (10^8 nm^3/s)	$k_{\mathrm{off,\ 3D}}$ (10^5 s^{-1})
6.65	15	100	2276.8 ± 22.1	3.10 ± 0.02	1.36 ± 0.01
6.65	15	10	2324.3 ± 22.6	3.10 ± 0.02	1.33 ± 0.01
6.65	14	10	711.1 ± 16.4	2.60 ± 0.04	3.67 ± 0.06
11.4	15	100	2306.6 ± 22.3	3.15 ± 0.02	1.37 ± 0.01
11.4	14	10	620.8 ± 15.2	2.42 ± 0.04	3.90 ± 0.06

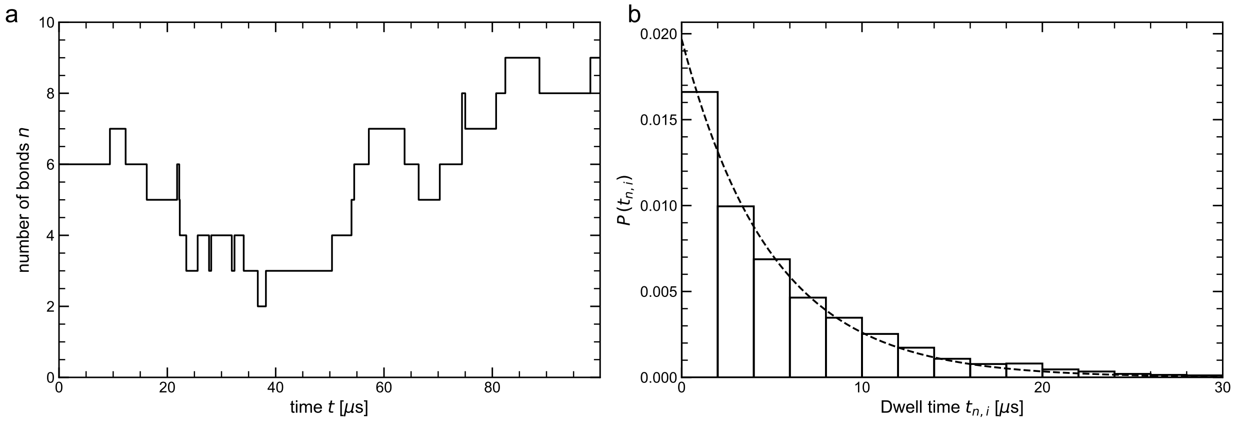

Figure S1: (a) Number of receptor-ligand bonds n as a function of time t for a short time interval of a simulation with 18 membrane receptors and 18 soluble ligands and membrane area 60×60 nm^2. (b) Dwell-time probability distribution for state $n = 3$, obtained from the same simulation system. The dashed line represents an exponential fit.

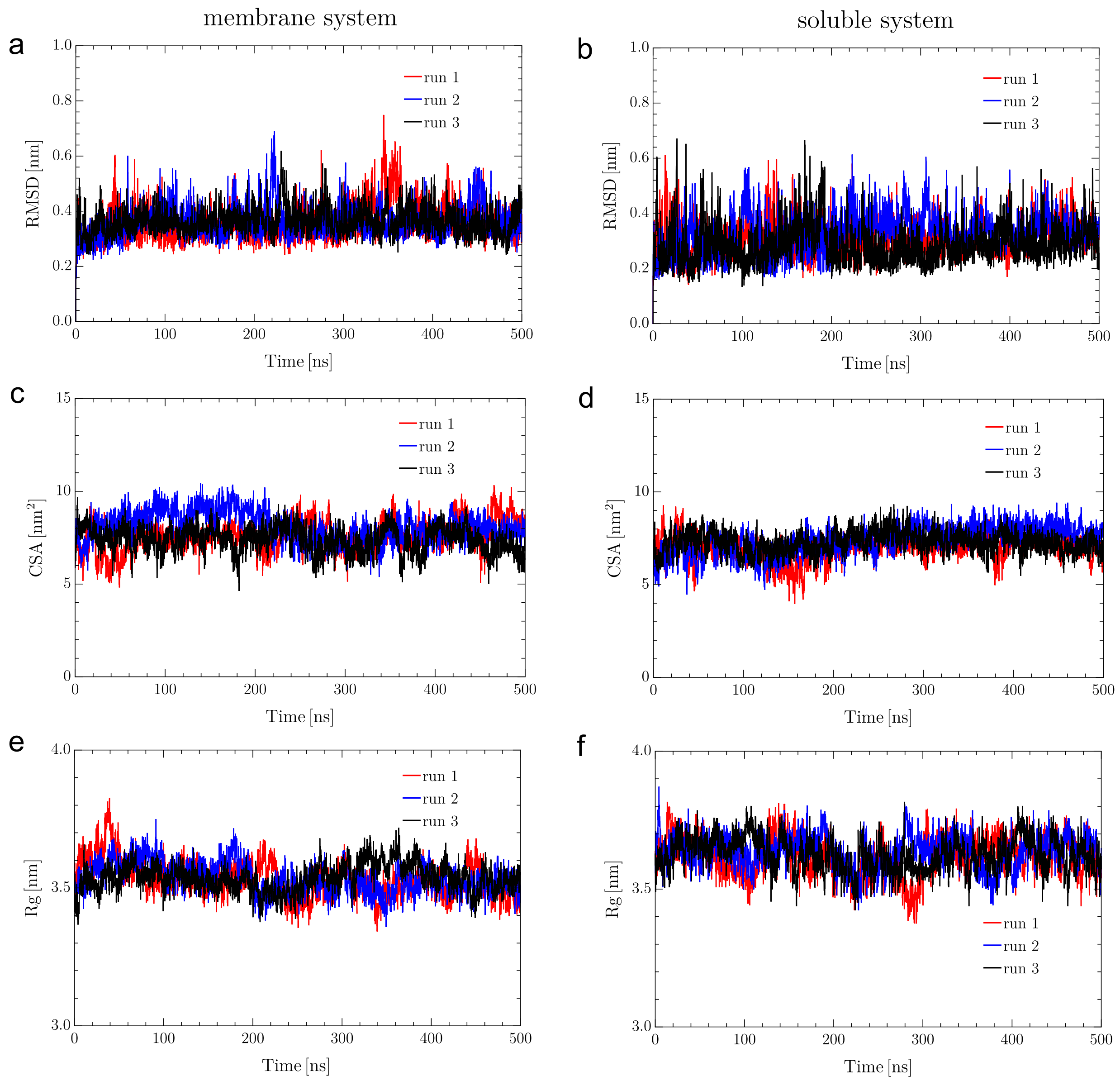

Figure S2: Results from AA-MD simulations of the CD2-CD58 complex in the membrane (a, c, e) and soluble (b, d, f) systems as illustrated in Fig. 4a. RMSD is the root-mean-squared deviation of the backbone atoms in the complex excluding the transmembrane domain (TMD) of CD2. CSA is the contact surface area between CD2 and CD58 in the complex. R_g is the root mean square radius of gyration of the complex without CD2's TMD. Each of the three independent runs starts from different initial equilibrated configurations.

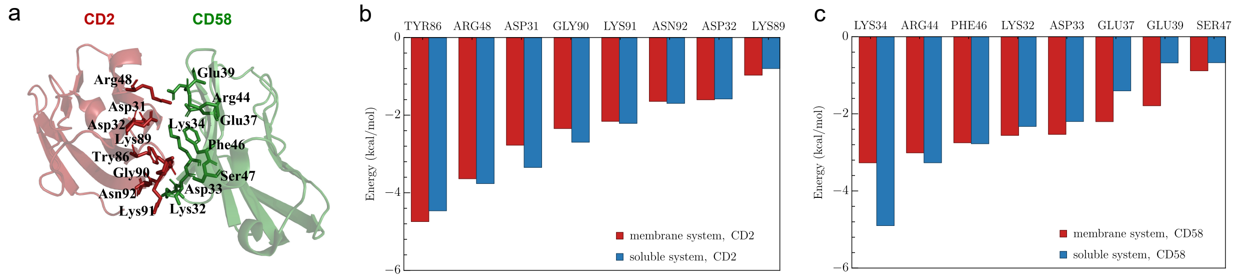

Figure S3: Key residues at the binding interface between CD2 and CD58 identified from energy decomposition. (a) Selected key residues. Energy per residue for the top eight key residues of CD2 (b) and CD58 (c) in soluble and membrane systems.

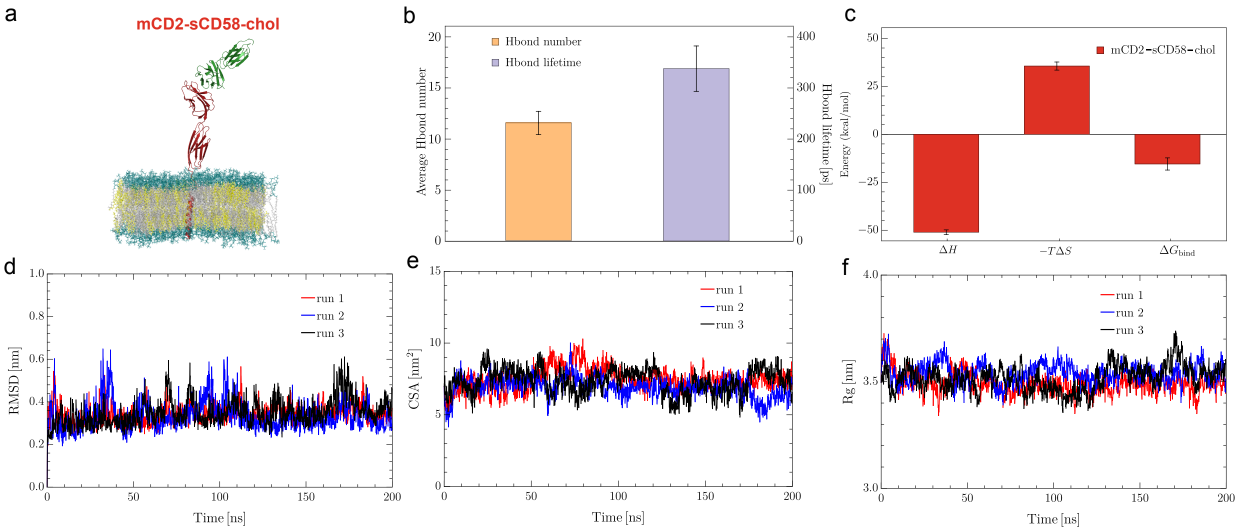

Figure S4: (a) Snapshot of the CD2-CD58 complex bound to a membrane composed of POPC (blue) and cholesterol (yellow) at a 7:3 ratio. (b) Average number and lifetime of hydrogen bonds between mCD2 and sCD58. (c) Decomposition of the binding free energy.(d) RMSD, (e) CSA, and (f) Rg of the CD2-CD58 complex over 200 ns simulations in three independent runs.

References

(1) Cooke, I. R.; Deserno, M. Solvent-free model for self-assembling fluid bilayer membranes: Stabilization of the fluid phase based on broad attractive tail potentials. *J. Chem. Phys.* **2005**, *123*, 224710.

(2) Dimova, R. Recent developments in the field of bending rigidity measurements on membranes. *Adv. Colloid Interface Sci.* **2014**, *208*, 225–234.

(3) Nagle, J. F. Introductory lecture: basic quantities in model biomembranes. *Faraday Discuss.* **2013**, *161*, 11–29.

(4) Zhu, Y.; Liu, H.; Li, Z.; Qian, H.; Milano, G.; Lu, Z. GALAMOST: GPU-accelerated large-scale molecular simulation toolkit. *J. Comput. Chem.* **2013**, *34*, 2197–2211.

(5) Abraham, M. J.; Murtola, T.; Schulz, R.; Páll, S.; Smith, J. C.; Hess, B.; Lindahl, E. GROMACS: High performance molecular simulations through multi-level parallelism from laptops to supercomputers. *SoftwareX* **2015**, *1*, 19–25.

(6) Xie, Z.-R.; Chen, J.; Wu, Y. Linking 3 D and 2 D binding kinetics of membrane proteins by multiscale simulations. *Protein Sci.* **2014**, *23*, 1789–1799.

(7) Maier, J. A.; Martinez, C.; Kasavajhala, K.; Wickstrom, L.; Hauser, K. E.; Simmerling, C. ff14SB: improving the accuracy of protein side chain and backbone parameters from ff99SB. *J. Chem. Theory Comput.* **2015**, *11*, 3696–3713.

(8) Tripathi, N.; Leherte, L.; Vercauteren, D. P.; Laurent, A. D. Structure-based identification of inhibitors disrupting the CD2–CD58 interactions. *J. Comput. Aided Mol. Des.* **2021**, *35*, 337–353.

(9) Bodian, D. L.; Jones, E. Y.; Harlos, K.; Stuart, D. I.; Davis, S. J. Crystal structure of the extracellular region of the human cell adhesion molecule CD2 at 2.5 Å resolution. *Structure* **1994**, *2*, 755–766.

(10) Ikemizu, S.; Sparks, L. M.; van der Merwe, P. A.; Harlos, K.; Stuart, D. I.; Jones, E. Y.; Davis, S. J. Crystal structure of the CD2-binding domain of CD58 (lymphocyte function-associated antigen 3) at 1.8-Å resolution. *Proc. Natl. Acad. Sci.* **1999**, *96*, 4289–4294.

(11) Wang, J.; Smolyar, A.; Tan, K.; Liu, J.; Kim, M.; Sun, Z.; Wagner, G.; Reinherz, E. Structure of a heterophilic adhesion complex between the human CD2 and CD58 (LFA-3) counterreceptors. *Cell* **1999**, *97*, 791–803.

(12) Fiser, A.; Sali, A. ModLoop: automated modeling of loops in protein structures. *Bioinformatics* **2003**, *19*, 2500–2501.

(13) Jo, S.; Kim, T.; Iyer, V. G.; Im, W. CHARMM-GUI: a web-based graphical user interface for CHARMM. *J. Comput. Chem.* **2008**, *29*, 1859–1865.

(14) Jorgensen, W. L.; Thomas, L. L. Perspective on free-energy perturbation calculations for chemical equilibria. *J. Chem. Theory Comput.* **2008**, *4*, 869–876.

(15) Shivakumar, D.; Williams, J.; Wu, Y.; Damm, W.; Shelley, J.; Sherman, W. Prediction of absolute solvation free energies using molecular dynamics free energy perturbation and the OPLS force field. *J. Chem. Theory Comput.* **2010**, *6*, 1509–1519.

(16) Virnau, P.; Müller, M. Calculation of free energy through successive umbrella sampling. *J. Chem. Phys.* **2004**, *120*, 10925–10930.

(17) Awasthi, S.; Kapil, V.; Nair, N. N. Sampling free energy surfaces as slices by combining umbrella sampling and metadynamics. *J. Comput. Chem.* **2016**, *37*, 1413–1424.

(18) Kästner, J.; Senn, H. M.; Thiel, S.; Otte, N.; Thiel, W. QM/MM free-energy perturbation compared to thermodynamic integration and umbrella sampling: Application to an enzymatic reaction. *J. Chem. Theory Comput.* **2006**, *2*, 452–461.

(19) Lin, Y.-L.; Aleksandrov, A.; Simonson, T.; Roux, B. An overview of electrostatic free energy computations for solutions and proteins. *J. Chem. Theory Comput.* **2014**, *10*, 2690–2709.

(20) Rocklin, G. J.; Mobley, D. L.; Dill, K. A.; Hünenberger, P. H. Calculating the binding free energies of charged species based on explicit-solvent simulations employing lattice-sum methods: An accurate correction scheme for electrostatic finite-size effects. *J. Chem. Phys.* **2013**, *139*.

(21) Chodera, J. D.; Mobley, D. L.; Shirts, M. R.; Dixon, R. W.; Branson, K.; Pande, V. S. Alchemical free energy methods for drug discovery: progress and challenges. *Curr. Opin. Struct. Biol.* **2011**, *21*, 150–160.

(22) Kollman, P. A.; Massova, I.; Reyes, C.; Kuhn, B.; Huo, S.; Chong, L.; Lee, M.; Lee, T.; Duan, Y.; Wang, W.; others Calculating structures and free energies of complex molecules: combining molecular mechanics and continuum models. *Acc. Chem. Res.* **2000**, *33*, 889–897.

(23) Genheden, S.; Ryde, U. The MM/PBSA and MM/GBSA methods to estimate ligand-binding affinities. *Expert Opin. Drug Discov.* **2015**, *10*, 449–461.

(24) Rastelli, G.; Rio, A. D.; Degliesposti, G.; Sgobba, M. Fast and accurate predictions of binding free energies using MM-PBSA and MM-GBSA. *J. Comput. Chem.* **2010**, *31*, 797–810.

(25) Wang, E.; Sun, H.; Wang, J.; Wang, Z.; Liu, H.; Zhang, J. Z.; Hou, T. End-point binding free energy calculation with MM/PBSA and MM/GBSA: strategies and applications in drug design. *Chem. Rev.* **2019**, *119*, 9478–9508.

(26) Valdés-Tresanco, M. S.; Valdés-Tresanco, M. E.; Valiente, P. A.; Moreno, E. gmx_MMPBSA: a new tool to perform end-state free energy calculations with GROMACS. *J. Chem. Theory Comput.* **2021**, *17*, 6281–6291.

(27) Hou, T.; Wang, J.; Li, Y.; Wang, W. Assessing the performance of the molecular mechanics/Poisson Boltzmann surface area and molecular mechanics/generalized Born surface area methods. II. The accuracy of ranking poses generated from docking. *J. Comput. Chem.* **2011**, *32*, 866–877.

(28) Chen, F.; Liu, H.; Sun, H.; Pan, P.; Li, Y.; Li, D.; Hou, T. Assessing the performance of the MM/PBSA and MM/GBSA methods. 6. Capability to predict protein–protein binding free energies and re-rank binding poses generated by protein–protein docking. *Phys. Chem. Chem. Phys.* **2016**, *18*, 22129–22139.

(29) Soderhjelm, P.; Kongsted, J.; Ryde, U. Ligand affinities estimated by quantum chemical calculations. *J. Chem. Theory Comput.* **2010**, *6*, 1726–1737.

(30) Nguyen, D. T.; Case, D. A. On finding stationary states on large-molecule potential energy surfaces. *J. Phys. Chem.* **1985**, *89*, 4020–4026.

(31) Hu, J.; Lipowsky, R.; Weikl, T. R. Binding constants of membrane-anchored receptors and ligands depend strongly on the nanoscale roughness of membranes. *Proc. Natl. Acad. Sci.* **2013**, *110*, 15283–15288.

(32) Hou, R.; Ren, S.; Wang, R.; Różycki, B.; Hu, J. Multiscale Simulations of Membrane Adhesion Mediated by CD47-SIRPα Complexes. *J. Chem. Theory Comput.* **2025**, *21*, 2030–2042.
